# Elderly patients with Alzheimer’s disease and their family relationships: Caregiver perspectives

**DOI:** 10.1590/S1980-57642011DN05020010

**Published:** 2011

**Authors:** Meire Cachioni, Thaís Bento Lima-Silva, Tiago Nascimento Ordonez, Juliana Galo-Tiago, Ana Regina Alves, Milena Yuri Suzuki, Deusivania Vieira da Silva Falcão

**Affiliations:** 1 Professor, PhD in Gerontology from the State University of Campinas, Campinas SP, Brazil and lecturer at the School of Arts, Sciences and Humanities of the University of São Paulo, São Paulo SP, Brazil. Coordinator of the Psychoeducational Intervention Group for Caregivers of Elderly patient with Alzheimer’s Disease from the Rehabilitation Center and Geriatric Day Care Hospital at the Institute of Psychiatry of the Clínicas Hospital of the USP School of Medicine, São Paulo SP, Brazil; 2 Graduate in Gerontology, PhD in Neurosciences from the ABC School of Medicine, Santo André Foundation, Santo André SP, Brazil and reading for Master’s in Neurology at the USP School of Medicine, São Paulo SP, Brazil; 3 Graduate in Gerontology, School of Arts, Sciences and Humanities of the University of São Paulo, São Paulo SP, Brazil; 4 Professor, PhD in Psychology from the State University of Brasília, Brasília DF, Brazil and lecturer at the School of Arts, Sciences and Humanities of the University of São Paulo, São Paulo SP, Brazil. Coordinator of the Psychogerontologic, Socio-familial and Educational Care Program for Caregivers and Relatives of Elderly with Alzheimer’s Disease from the Rehabilitation Center and Geriatric Day Care Hospital at the Institute of Psychiatry of the Clínicas Hospital of the USP School of Medicine, São Paulo SP, Brazil

**Keywords:** elderly, dementia, Alzheimer’s disease, family relationships

## Abstract

**Objective:**

The present study was to investigate the status of the family relationships of elderly patients with Alzheimer’s disease from a caregiver’s perspective.

**Methods:**

A total of sixteen relatives/caregivers of AD patients were assessed by applying a semi-structured questionnaire about family relationships. Frequency tables containing categorical variables (gender, schooling, personal income and current occupation) were compiled. Descriptive statistics were calculated of continuous variables such as age. Answers to open questions were submitted to Bardin’s content analysis. The data were held on the Epidata program and statistical analysis was performed using the SPSS v.17.0 software package.

**Results:**

In line with the literature, the family was the main source of caregivers and the typical caregiver profile was female. In contrast to other studies, emotional burnout was not a major complaint in the sample studied. The ties created among family members and the life experience of each individual influences how interviewees cope with AD.

**Conclusion:**

The family relationships of caregivers of AD patients represent a constant challenge, due to changes in roles within the family structure as well as to disease progression. However, participation in psychological and socio-educational activities run by pioneering institutions in Brazil, act as a mediator of stress in the lives of both caregivers and patients.

## Abstract (pt)

**Objetivos:**

Buscou-se no presente estudo averiguar como ocorrem as relações familiares de idosos portadores de Alzheimer na perspectiva de cuidadores.

**Métodos:**

Participaram do estudo dezesseis familiares/cuidadores, aplicando-se roteiro semi-estruturado sobre relações familiares. Foram feitas tabelas de frequência das variáveis categóricas (gênero, escolaridade, renda individual e ocupação atual). Foram calculadas estatísticas descritivas das variáveis contínuas, como a idade. As respostas às questões abertas foram submetidas à análise de conteúdo de Bardin. Utilizou-se o programa Epidata para armazenamento dos dados e para análise estatística o programa computacional SPSS v.17.0.

**Resultados:**

A família é a principal fonte de cuidado, o perfil do cuidador em sua maioria foi do gênero feminino e a sobrecarga emocional diferentemente dos estudos encontrados não foi uma queixa muito presente na amostra estudada. Considera-se que os vínculos criados entre os membros familiares e a experiência de vida de cada indivíduo influenciam na maneira como os entrevistados lidam com a DA.

**Conclusão:**

As relações familiares de cuidadores de pacientes com DA é um desafio permanente, resultante tanto da troca de papéis na estrutura familiar, como em virtude da progressão da doença do paciente. Entretanto a participação em atividades psicológicas e socioeducativas em instituições pioneiras no Brasil atuam como mediadores do estresse na vida tanto do cuidador como do paciente.

The feeling of belonging to a family involves affection, freedom, reciprocity, shared stories, in other words, aspects inherent to the condition of being human that touch on conscious and unconscious issues.^1^ Understanding family relations first requires being aware of the significance of these relationships in one’s life and above all, identifying the connotation of the word family in our society.

The family is not a natural occurrence but a cultural achievement, being deep-rooted in a history of creation and transformation.^1^ In terms of its structures and functions, the term family has been used to designate different social institutions and groupings. These organs were not necessarily concerned with everyday or generational reproduction as a specific or exclusive function, at times playing primarily political and economic roles.

It is important to clarify that family is not exclusively a single and inflexible nucleus of a psychological, sociological and anthropological nature, as decades-old theories have described. Falcão^1^ conceived family as a tight network of personal ties which provide a sense of belonging and group with limits, ideals, rules and modes of communication which may or may not differentiate interindividual human relationships. To this day, the family unit constitutes the main source of support at times of need.^2^

On this note, studies involving caregivers are relatively recent in the literature. Studies on caregivers of psychiatric patients first emerged in the 1960s, followed by studies on caregivers of frail elderly, and in the 1980s and 1990s by research on caregivers of demented elderly, including patients with Alzheimer’s disease.^2^

Studies have revealed that around 40% of individuals aged 65 years or older need some kind of assistance with at least one task, such as shopping, handling finances, preparing meals or doing the cleaning. According to Karsh,^3^ a lesser proportion (10%) require help to perform basic tasks such as taking a bath, getting dressed, using the restroom, feeding oneself, or getting in and out of chairs and beds. These data reflect concerns of more 6 million individuals and families, and the one and a half million highly dependent elderly in Brazil, based on information from the 2001 National Household Sampling Survey (PNAD).^4^

However, the concept of “caregiver” remains the subject of intense debate, with no consensus having been reached over its definition, interfering in studies on the act of caregiving.^5^ According to Leitão and Almeida,^6^ the caregiver is an individual who assumes responsibility for caring, provides support, or assists in a need of the cared person, in an effort to improve their health. Nascimento^7^ defined a caregiver as an individual who provides care to cater for temporary or permanent functional disability. Another question that is the focus of debate relates to the factors designating the caregiver type needed by each dependent elder, and to attempts to classify caregivers into informal, formal, primary or secondary categories.^3^ According to Sommerhalder,^8^ formal caregivers encompass all professionals and institutions that render care in the capacity of service providers, whereas informal caregivers include family members, friends, neighbors, fellow church members, etc. With regard to the distinction between primary and secondary caregivers, the frequency of care and degree of involvement are determinants, whereby primary caregivers are considered those who have full or greater responsibility for care provided in the home, while secondary caregivers perform activities considered supplementary to those of the primary caregiver.^9^

According to Karsch,^3^ four factors are considered in designating those who take on the personal care of a debilitated elderly person: kinship (spouses), gender (predominantly women), physical proximity (live together), and affective proximity (spouse, parents and children). The gerontological literature shows that, in the vast majority of Western countries, the care-related tasks are generally attributed to women. Since women are more long-lived than men, and generally younger than their husbands, the preferential family caregiver is the wife. In the absence of a wife, the second generation descendents tend to take on the task of caregiving.^10^

Besides, generational, gender and kinship factors influencing the attribution of the task of caregiving, other aspects such as living under the same roof, having the financial means and time available, are common determinants of eligibility as caregiver. In addition, affective ties, the caregiver’s personality, past relationship with the elderly person, the reason and ability to donate, also affect availability as caregiver.^11^

Nevertheless, it should be noted that although having a caregiver who is a relative may be important, this is not relevant for all elderly since many older adults have no family. On the other hand, some elderly come from very poor families whose members are unable to give up their jobs to take on the role of caregiver. Such situations can exacerbate morbidity.^12^

In view of the peculiarities of Alzheimer’s disease, the caregiver performs a key role since they are involved in virtually all aspects of care, taking on a progressively increasing number of responsibilities. In other words, as the disease progresses, the patient needs ever more help to carry out instrumental activities of everyday life, such as managing finances and medicines. At more advanced stages of disease, caregivers are needed to provide assistance with more basic activities of daily living, such as personal hygiene, bathing and feeding. Caregivers dedicate, on average, 60 hours per week to performing this task, making the role of caregiver a contributory factor to burnout.^12^

In this scenario, the importance of the work by multiprofessional team in maintaining the quality of life of both the elderly and their family becomes evident. The aim of the present study was to ascertain the status of the family relationships of patients with Alzheimer’s disease and other dementias, and to verify the meaning of these relationships from the standpoint of formal and familial caregivers.

## Methods

### Participants and study type

A study with a qualitative, exploratory, and descriptive design was performed which sought to investigate the experiences of the family members that care for elderly relatives with Alzheimer’s disease. The study sample included formal and informal caregivers who frequented support programs for family and caregivers of patients with Alzheimer’s disease. Participants had taken part in psychoeducational interventions run in June 2009 by support institutions for dementia patients, including individuals with Alzheimer’s disease.

### Study site

The present study was performed at two support institutions for elderly with Alzheimer’s disease and their relatives and/or caregivers: the Brazilian Association of Alzheimer’s or Similar Diseases (ABRAz); and the Geriatric Day Care Hospital Program - Psychiatry Institute - Hospital das Clínicas. The latter runs a program called the Rehabilitation Center and Geriatric Day Care Hospital at the Institute of Psychiatry (IPq) of the Hospital das Clínicas (USP School of Medicine - HCFMUSP) which selects patients over the age of 60 in the initial phase of Alzheimer’s disease, who are undergoing treatment with medicines. Patients selected took part in a program offering cognitive and functional stimulation activities, in conjunction with pharmacological treatment.^14^

This program also offers two activities aimed at formal and informal caregivers, namely, “Psychoeducational Intervention Group for Caregivers of Elderly with Alzheimer’s Disease” and the “Psychogerontologic and Socioeducational Care Group for family members and caregivers”, both of which aim to orient individuals involved in caregiving on the process and demands related to the disease. The programs provide a forum in which participants can share experiences. Each group meets on a weekly basis, where activities are run over a series of 30 sessions.^13^

### Instruments

Prior to conducting the interviews, all participants were given an Informed and Free Consent Term. This was followed by a letter of presentation about the interviewers. After participants had formally given their consent, the interview was conducted by applying a semi-structured questionnaire containing both open and closed questions to family members and caregivers.

### Procedures

After selecting the institutions, initial contact was made with the administrators of each unit. The interviews were carried out after (or before) the performing of socio- and psycho-educational activities, under the coordination of psychologists and with participation of Gerontology undergraduate interns.

### Statistical analysis

Frequency tables of categorical variables (gender, schooling, personal income and current occupation) were built to provide a profile of the sample. Descriptive statistics were calculated (with measures of position and dispersion: mean, standard deviation, minimum and maximum values) of continuous variable such as age. The data were held on the Epidata program and statistical analysis was performed using the SPSS v.17.0 software package.

Bardin’s analysis theory was employed to perform content analysis of participants’ responses to the open questions.^16^ Based on this theory, content analysis aims to obtain a description of the content of the messages and indicators, which may or may not by quantitative, to enable the inference of knowledge regarding the production and reception of these messages.

Thus, content analysis is generally done by frequency deduction or analysis by thematic category. Frequency deduction entails performing a count of the occurrences of the same linguistic sign (word) which frequently repeat, and numeric descriptions and statistical analysis based on it. Analysis by thematic category on the other hand, is based on judging the codifier, which identifies meanings and characterizes them into one of the equivalent classes.

Content analysis involves three steps:

[1] pre-analysis, which entails organizing the content collected by means of floating reading, hypotheses, objectives and devising of indicators on which to base interpretation;
[2] exploitation of the content, in which the data are codified from registration units; and
[3] treatment of results and interpretation, in which categorization takes place, entailing classification of elements according to similarity and by differentiation, with subsequent regrouping in the event of common characteristics.^16^

## Results

The majority of caregivers had been caring for the elderly patient since the onset of their disease. However, some children reported having always taken care of their parents by helping on money matters for instance. It was evident that, overall, the members of the family are the first to be contacted to help in caring for the elder. Indeed, the family predominates as an option in the informal support system for elderly (Charts 1 and 2).

**Chart 1:** How long have you been responsible for the care of this elder?

Categories	Examples	n=17	%
Always	- I've always took care of them (informal caregivers)	3	17.66
Since disease onset	My mother has been the caregiver since the AD diagnosis. Ever since the disease emerged about six years ago. Since she fell ill, it's my husband I had been her caregiver since she was widowed but she lived in Ribeirão Preto. She's been living with me for four years, ever since her disease was diagnosed.	4	23.53
For one year	Since 2007, one year ago.	1	5.88
For seventeen years	Since 1991, they died recently.	1	5.88
For a few months	Since early 2008, a few months ago.	3	17.65
For twenty-nine years	For twenty-nine years.	1	5.88
For some years	Three years and four months ago. One and a half years ago.	2	11.76
Answer not given		2	11.76

**Chart 2:** Generally, who do you turn to when you need help?

Categories	Examples	n=17	%
Nobody	Nobody	2	11.76
Family	I turn to my granddaughter I turn to my sister, friends and therapists .Her daughter. I seek the daughters or, depending on the situation, the husband. I turn to my family members.	11	64.70
Domestic	I get help from the domestic.	1	5.89
Professional assistance	I turn to my sister, friends and therapists .I go to the family and the doctors for guidance.	2	11.76
Answer not given		1	5.89

Of the caregivers of AD patients interviewed, 31% reported they were caring for the elder satisfactorily, while 25% stated they were fatigued. Results also showed that from the perspective of caregivers and family members of elderly with Alzheimer’s disease, the main role of the family is as caregiver. Most of the caregivers interviewed reported they had some kind of formal or informal support. The most frequent response was that the family was the provider of the majority of the support (50% frequency). Changes in the relationship between caregivers or family members and elderly following the onset of Alzheimer’s disease, were noted (Charts 5 and 6).

**Chart 5:** Apart from your assistance, does the elder get any other kind of help (financial or emotional) from other members of the family? How often?

Categories	Examples	n=18	%
No	No	4	22.22
Family members	Emotional support from other siblings, and financial from another sister who lives with them.Yes, she gets emotional and financial support from the daughters and husband.	9	50
Yes	Yes.	2	11.11
Priest	Weekly, fortnightly and monthly. Emotional and financial support.	1	5.55
Frequently	Frequently receives help.	2	11.11

**Chart 6:** How was your relationship with the elder with AD prior to the disease and what is it like now?

Categories	Examples	N=16	%
Unchanged.	- We were, and continue to be, close. My relationship has always been the best possible.	7	43.75
Changed	It was normal but now it's more aggressive. It was more distant and colder because he worked away from home After AD he stopped working and we became closer. I also give him more attention.	8	50
Did not know prior to AD.	Did not know them before the disease.	1	6.25

## Discussion

Brazilian epidemiological studies report that approximately 7% of elderly meet criteria for dementia,^17^ while 16% present with some degree of cognitive and/or functional decline.^18^ These studies indicate that cognitive decline, as well chronic diseases, represent an important public health issue, in virtue of the dependence and family burden they cause.

Against this background, the present study sought to ascertain the status of family relationships of elderly dementia patients. The study findings corroborated data in the literature indicating that women are the predominant caregivers for the elderly. This constitutes a secular female duty. Women are more long-lived and generally younger than their husbands.^19^ Spouses were most often the caregivers, given the marriage bond. The social norms of caring for the elderly show that the wife tends to head the hierarchy, followed by the eldest daughter, and then widowed or single daughters. On this point, studies have shown that male caregivers are the exception since it is the man who is the breadwinner and the moral head of the family.^20^

However, studies confirm that in fact, the caregiver is often the person most available and best prepared. The choice of caregiver tends to be based on generational, gender and kinship factors as well as living under the same roof, having the financial means and time available, and emotional attachment, congruent with the findings of the present study. In this context, the Spanish Society of Geriatrics and Gerontology^21^ has reported that, in most cases, the caregiver of the elderly in Spain are married, older adults, and both lend assistance in caring for the elder. Moreover, the fact that these caregivers are not in paid employment, the absence of care support and the substitution of their role as caregiver by other family members, rarely takes place.

However, the study by Santos^22^ reveals not a main caregiver figure, but rather a network of caregivers (members of the church which they belong to, home domestics etc.). The family dynamic shifts over the course of the disease, such that the roles of the provider, decision-making and power are taken over by other members of the family. Alzheimer’s disease affects the whole family nucleus and not only the dementia patient. The disease leads to a family restructure and, as the disease progresses, greater care is required.^23^ Families change the *modus operandi* in order to reorganize, by rotating the tasks of caring for instance. At this point, early conflicts resurface and there may be resistance to assuming the roles, which in turn affects the quality of life of the elder as well as that of their family members.^1^

Based on the results of the present study, it is clear that from the perspective of caregivers and family members of elderly with Alzheimer’s disease, the main role of the family is as caregiver. Similarly, Fonseca and Soares^23^ affirmed that is was common for society to hold the family responsible for providing care to the elderly who have become dependents in some way. This stance on elderly and their family members often boils down to a question of culture, since most societies consider intergenerational interaction as the mainstay of their culture. According to Lima and Marques,^24^ 80% to 90% of care is provided by family members of the elderly, and ranges from nursing and medical care, and personal services, to help in carrying out activities of daily living.

Another point considered equally important in the present study was categorized as union, being cited by 5 respondents. Lima and Marques^24^ believe that family relationships are strongly underpinned by emotions and feelings, such as affection, love, union, responsibilities and moral and ethical duties.

Corroborated by our findings, studies such as that by Cohen et al.^25^ have reported that informal caregivers see more positive aspects in the task of caregiving. The cited study involving caregivers of dementia patients in Canada showed that companionship, fulfillment, satisfaction, providing quality of life, and having a purpose or aim in life, were feelings that stood out in relation to the burden of caregiving. These positive aspects are experienced when the caregiver perceives that the task of caring is being fulfilled and the elder recognizes the assistance they are being given. However, tension, stress, compromised physical state, psychological disorders, helplessness and financial difficulties may also affect caregivers.^8^

Interviewees in the present study also reported that the family was important in caring for AD patients. Lima and Marques^24,27^ highlighted that the family was deemed the most important source of support to the elderly, since they assisted both financially and in the provision of care in the event of illness.

Comfort and security were also cited by the participants in the present study, suggesting that the presence of the family provides both comfort and a sense of security for family members and elders alike. Neri^26^ affirmed that the familial caregiver who assumes responsibility for the elder in terms of emotional and cognitive impairments also takes responsibility for their protection and maintenance.

Of those interviewed, only one cited that the role of the family is Acceptance and Patience, whereas according to the ABRAz,^27^ family members and caregivers appreciate that the role of the family, besides providing care, is to give the patient a sense of well-being. Caring for an individual with AD can be a challenging task, calling for patience, dedication and above all, assistance in the form of task-sharing amongst family members, as well as with a team of multi-professionals. This is necessary because patients need full-time care, generating physical and emotional fatigue among those who are directly involved with the patient. Support networks for caregivers are needed, especially in the social and health areas, such as guidance on homecare of elders, technical support linked to the health area which carers can turn to in the event of doubts and follow-up, all of which are often unavailable.^22^ Perracini and Neri^28^ carried out studies showing that, for middle-aged women and elders caring for dependent elderly, the existence of a social support network was essential for maintaining psychological and physical well-being.

Patient interventions are aimed at maintaining the abilities which are still preserved in the individual, in a bid to achieve the optimal functional state possible at each stage of the illness, reducing the decline inherent to the disease. The treatment of AD entails the use of pharmacological agents and multi- and inter-professional psychosocial and educational interventions aimed at reducing the impact of the problems arising from the disease on the lives of patients, caregivers and their families.

According to SAAD,^29^ the individuals giving care are most often the relatives who live in the same abode (80% of cases), followed by the children who do not live with the patient (around 49%). Shifts in support over to other relatives and non-relatives are less frequent, a phenomenon exemplified in the present study by a priest acting as caregiver for an AD patient.

Studies in the literature indicate that many caregivers and/or relatives receive no help or support from other members of the family, which could be explained by demographic changes or structural changes in families. However, some studies point to an urgent need for reform in geriatric healthcare services.^12,15^ Families are becoming increasingly nuclear, with a substantial shift in roles played by family members, who find it increasingly difficult to meet healthcare needs, potentially contributing to greater vulnerability among more severely debilitated elderly.^19^

In summary, the aim of this study was to investigate the relationships between family/caregiver and AD patients through institutions providing support programs for family members/caregivers. The results point to the fact that the majority of caregivers are informal (children and spouses). Most of those interviewed were married. The family is the main nucleus providing care and serving as a support network. A broad spectrum of sentiments was expressed by caregivers, such as giving back, fatigue, feeling good about the act of caregiving, concern, unhappiness and responsibility. The findings of the present study proved similar to data reported in the literature.

Although the main aim of this study was not to assess the effectiveness of the psychoeducational program for caregivers and dementia patients, reports by the participants showed that frequenting the interventions of the institutions changed the way some felt as providers of daily care to the patients. On this point, data in the literature suggest that psychoeducational interventions may provide extensive therapeutic benefits, leading to a substantial improvement in the subjective well-being and outlooks on life of individuals with dementia syndromes as well as caregivers and family members. Encouraging participants to make decisions, to reduce emotional overload and improve activities of caregiving to the elderly, contributes to a more amenable family atmosphere and to greater quality of care for patients and caregiver alike.^22,28,29^

To conclude, the present study contributes to the gerontological literature by providing new insights on the family relationships of formal and informal caregivers of patients with Alzheimer’s disease who take part in psychoeducational activities.

However, the study has several limitations including the fact that pre and post testing assessments could have been conducted in order to verify the impact of the psychoeducational interventions on biopsychosocial aspects of participants. Secondly, the use of a control group would also have been beneficial to better describe the impact of psychoeducational interventions on the care provided to the dementia patients.

Future longitudinal studies should be conducted in order to associate length of time participating in socioeducational programs and perceived quality of family relationships. Also, further studies could compare sociodemographics before and after evolution of the disease in order to determine which factors in the life of the caregiver are most impacted. This would yield valuable information to enable public policies and more interventions to be implemented in the community.

## Figures and Tables

**Table 1:** Sociodemographic profile of caregivers.

Variable	N=16	Mean and SD	%
Institution			
FMUSP	10		62.50
ABRAZ	6		37.50
			
Caregiver type			
Informal	12		75.00
Formal	4		25.00
			
Caregiver/Patient relationship			
Spouses	4		25.00
Children	7		43.80
Others (friend, formal caregiver)	5		31.30
			
Lives with elder?			
Yes	10		62.50
No	6		37.50
			
Gender			
Female	13		81.25
Male	3		18.75
		58.69 (12.53)	
Age			
20 < x ≤30	1		6.25
40 < x ≤50	1		6.25
50 < x ≤60	8		50.00
60 < x ≤70	3		18.75
70 < x ≤80	3		18.75
		10.50 (3.88)	
Schooling			
Primary school education (not concluded)	2		12.50
Primary school education (concluded)	5		31.25
High school education (not concluded)	1		6.25
High school education (concluded)	2		12.50
University level education (not concluded)	2		12.50
University level education (concluded)	4		25.00
			
Single	3		18.75
Married	10		62.50
Divorced	2		12.50
Widow(er)	1		6.25
			
Retired			
Yes	9		56.25
No	7		43.75
			
Working			
Yes	6		37.50
No	10		62.50
			
Personal income			
From 1 to 2 min. wages	2		12.50
From 2 to 3 min. wages	3		18.75
From 3 to 4 min. wages	0		0.00
From 4 to 5 min. wages	2		12.50
From 5 to 10 min. wages	1		6.25
Over 10 min. wages	6		37.50
Answer not given	2		12.50

**Chart 3:** How do you feel about caring for the elder?

Categories	Examples	N=16	%
I feel good	Good. It was hard at first, I was more fraught and worried. I'm more patient these days after having taken part in the support activity. I feel very useful.	5	31.25
Giving back	I feel as if I'm paying back everything good she did for me.	1	6.25
Responsibility	It's a big responsibility.	1	6.25
Fatigue and stress	Very tired and stressed	4	25
Unhappy	I feel anguished and unhappy for lack of improvements	1	6.25
Worried	I get really worried.	1	6.25
Answer not given		3	18.75

**Chart 4:** What's the meaning of family and what's their role regarding demented elderly ?

Categories	Examples	n=18	%
Caregivers	The family has to take care of its elderly members with AD, just family.	5	27.78
Important	The family is the foundation, pivotal, it's everything. Their role concerning elderly with AD is extremely important	3	16.67
Acceptance and patience	Acceptance and patience.	1	5.56
Union	The family is the union of people with blood ties. Their role is to provide attention and help in caring for elderly with AD	5	27.78
Comfort and security	Family is the life of the elder. They dedicated themselves and built a family; the family must now provide a little comfort and security.	1	5.56
Answer not given		3	16.67
